# Supplementary material for: Simultaneous Biological Pretreatment and Saccharification of Rice Straw by Ligninolytic Enzymes from Panus neostrigosus I9 and Commercial Cellulase
Source: J Fungi (Basel). 2021 Oct 12;7(10):853. doi: 10.3390/jof7100853 (PMC8537424; doi:10.3390/jof7100853)
Supplement: Supplementary file 1 [file jof-07-00853-s001.zip › jof-1394578-supplementary.pdf]

Journal of *Fungi*

Supplementary Material

Simultaneous Biological Pretreatment and Saccharification of Rice straw by *Panus neostrigosus*

Ariyah Terasawat [1], and Sivawan Phoolpundha [1,*]

[1] *Department of Microbiology Faculty of Science King Mongkut's University of technology Thonburi 126 Pracha-Uthid Road, Bang Mod, Thrung khru, Bangkok 10140, Thailand; Ariyah.tera@mail.kmutt.ac.th; sivawan.pho@kmutt.ac.th*
* Correspondence: *sivawan.pho@kmutt.ac.th*; Tel.: +662-4708887

Citation: Terasawat, A.; Phoolphundh, S. Simultaneous Biological Pretreatment and Saccharification of Rice straw by *Panus neostrigosus*. *J. Fungi* **2021**, *7*, 853. https://doi.org/10.3390/jof7100853

Academic Editor: Laurent Dufossé

Received: 9 September 2021
Accepted: 5 October 2021
Published: 12 October 2021

Publisher's Note: MDPI stays neutral with regard to jurisdictional claims in published maps and institutional affiliations.

Copyright: © 2021 by the author. Licensee MDPI, Basel, Switzerland. This article is an open access article distributed under the terms and conditions of the Creative Commons Attribution (CC BY) license (http://creativecommons.org/licenses/by/4.0/).

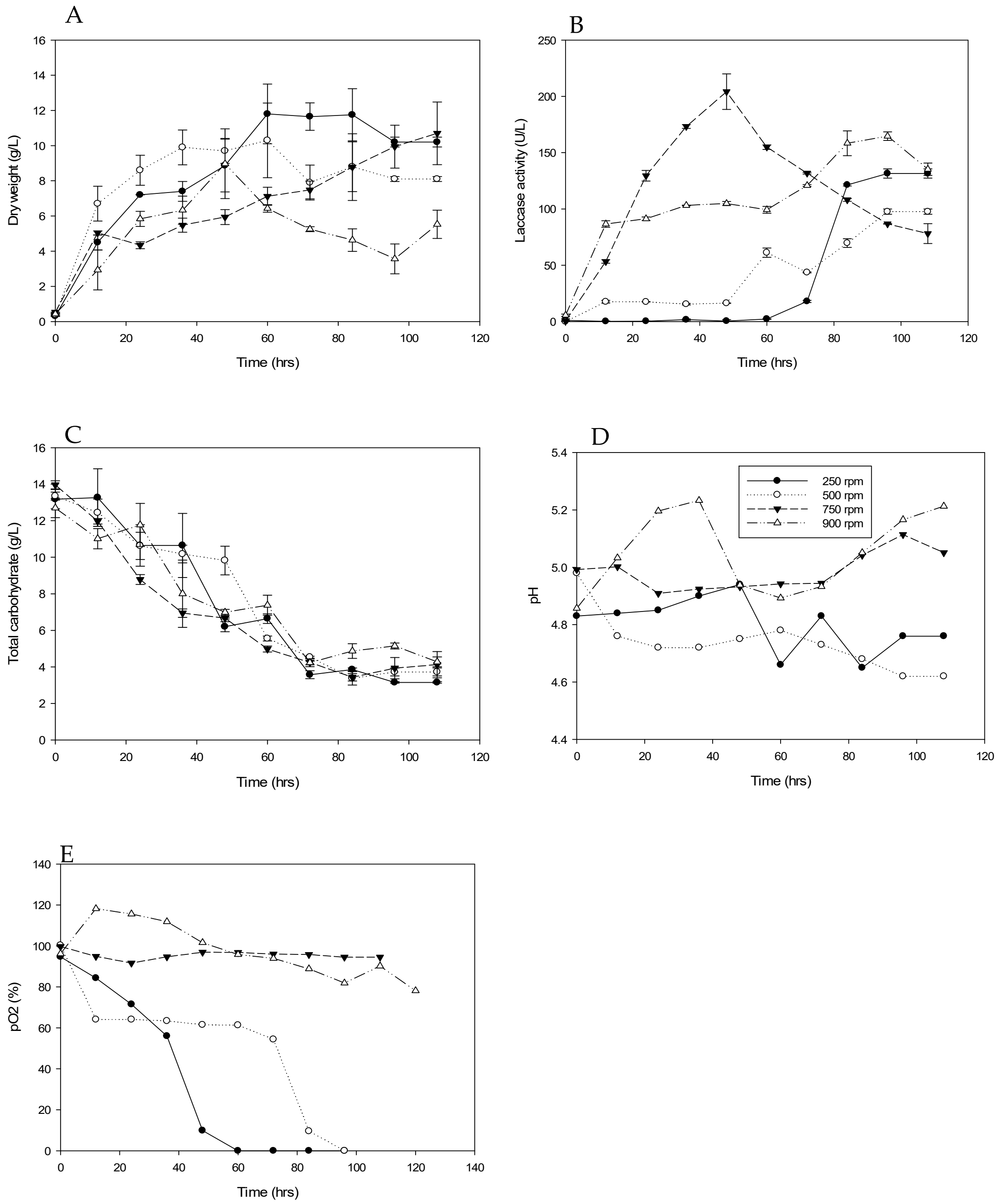

Figure S1. Time course of dry weight)A(, laccase activity)B(, total carbohydrate)C(and pH)D(and dissolved oxygen (E) during *P. neostrigosus* I9 cultivation in 2 liters STR at 28°C, aeration 1 vvm, at various agitation speeds. Values are average of results from triplicate trials; error bars indicate the SD values.

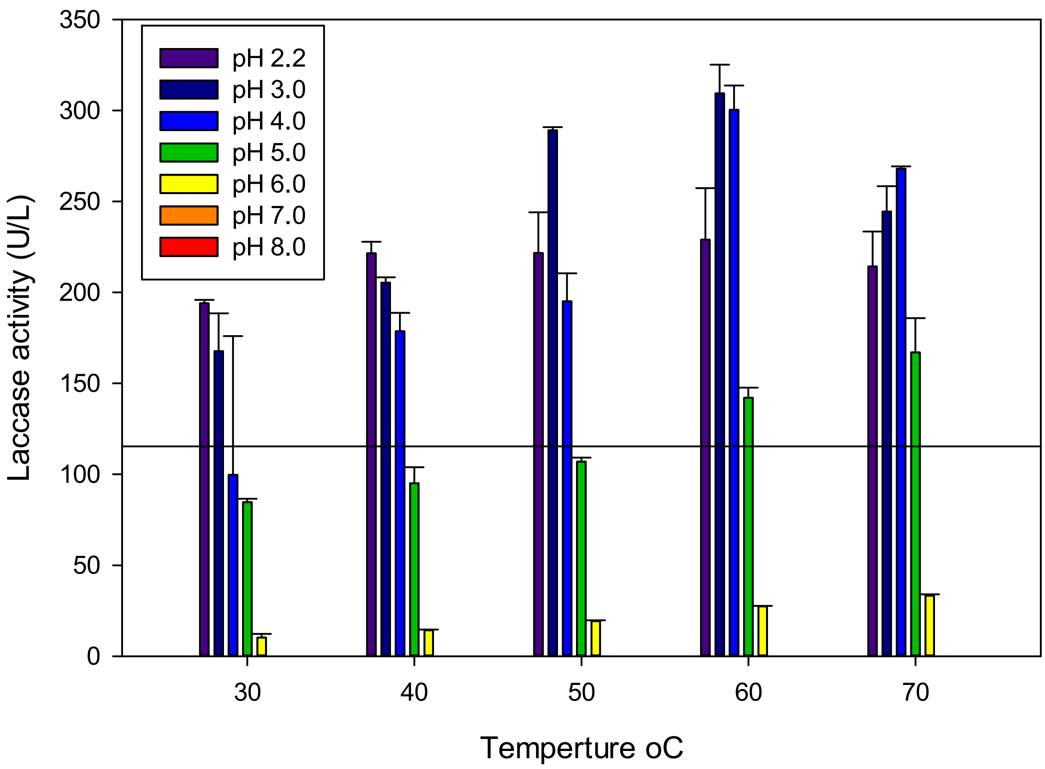

Figure S2. Effect of pH and temperature on laccase activity. Values are average of results from triplicate trials; error bars indicate the SD values (no laccase activity was detected in pH 7.0 and 8.0.)

A

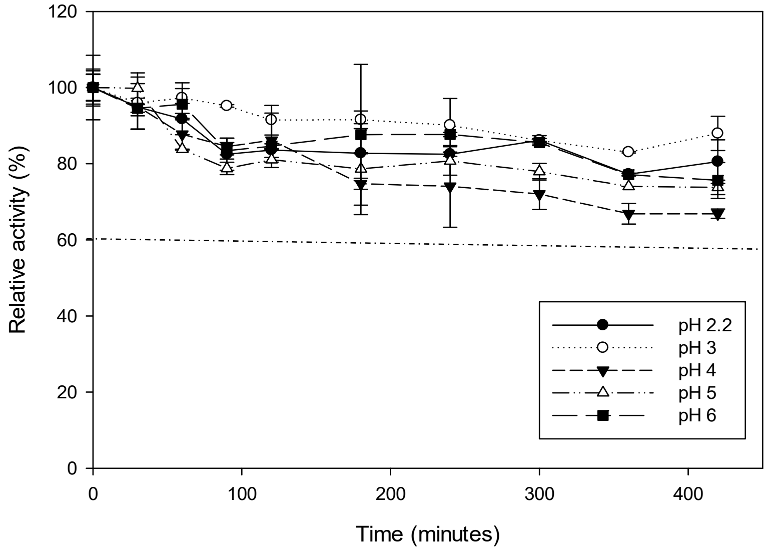

B

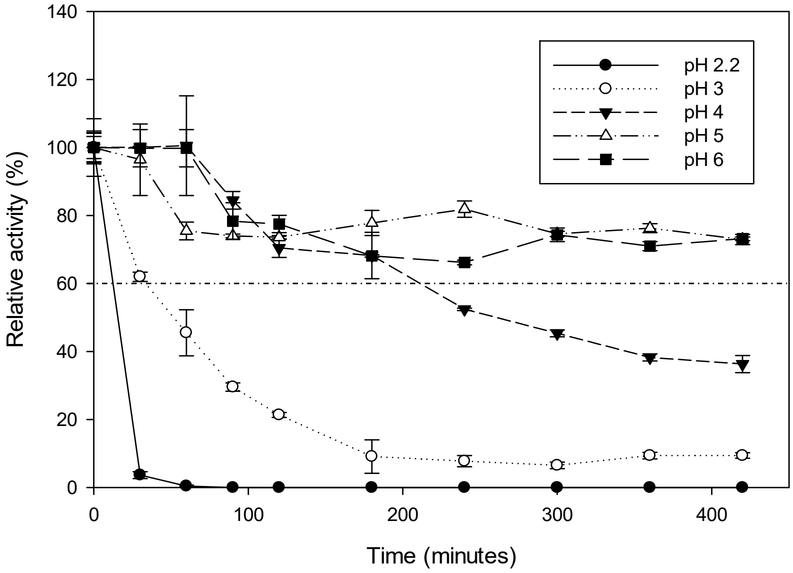

Figure S3. Comparison of relative laccase activity as function of pH at 30°C (A) and 60°C (B). Values are average of results from triplicate trials; error bars indicate the SD values.
